# Optimizing the modulation paradigm of transcutaneous auricular vagus nerve stimulation in patients with disorders of consciousness: A prospective exploratory pilot study protocol

**DOI:** 10.3389/fnins.2023.1145699

**Published:** 2023-03-15

**Authors:** Weihang Zhai, Haoyang Jiao, Yutong Zhuang, Yi Yang, Jinling Zhang, Yifei Wang, Yu Wang, Ya-nan Zhao, Shuai Zhang, Jianghong He, Peijing Rong

**Affiliations:** ^1^Institute of Acupuncture and Moxibustion, China Academy of Traditional Chinese Medicine, Beijing, China; ^2^Institute of Documentation, China Academy of Traditional Chinese Medicine, Beijing, China; ^3^Department of Neurosurgery, The Second School of Clinical Medicine, Southern Medical University, Guangzhou, China; ^4^Department of Neurosurgery, Beijing Tiantan Hospital, Capital Medical University, Beijing, China

**Keywords:** disorders of consciousness, transcutaneous auricular vagus nerve stimulation, minimally conscious state, coma recovery scale-revised, electroencephalography

## Abstract

**Background:**

Transcutaneous auricular vagus nerve stimulation (taVNS) is a non-invasive neuromodulation technique. Several studies have reported the effectiveness of taVNS in patients with disorders of consciousness (DOC); however, differences in the modulation paradigm have led to inconsistent treatment outcomes.

**Methods/design:**

This prospective exploratory trial will include 15 patients with a minimally conscious state (MCS) recruited according to the coma recovery scale-revised (CRS-R). Each patient will receive 5 different frequencies of taVNS (1, 10, 25, 50, and 100 Hz); sham stimulation will be used as a blank control. The order of stimulation will be randomized, and the patients’ CRS-R scores and resting electroencephalography (EEG) before and after stimulation will be recorded.

**Discussion:**

The overall study of taVNS used in treating patients with DOC is still in the preliminary stage of exploration. Through this experiment, we aim to explore the optimal stimulation frequency parameters of taVNS for the treatment of DOC patients. Furthermore, we expect to achieve a stable improvement of consciousness in DOC patients by continuously optimizing the neuromodulation paradigm of taVNS for the treatment of DOC patients.

**Clinical trial registration:**

https://www.chictr.org.cn/index.aspx, identifier ChiCTR 2200063828.

## Introduction

Following severe brain injury, consciousness can remain impaired for a long time, resulting in DOC (Wu et al., 2022), which can severely affect patients’ quality of life, placing a huge burden on society and their families. DOC includes coma, unresponsive wakefulness syndrome/vegetative state (UWS/VS), and minimally conscious state (MCS) (Gosseries et al., 2014). In contrast to patients with UWS/VS, patients with MCS exhibit signs of recovery of consciousness, such as visual pursuit, object localization, or following verbal commands (Giacino et al., 2002).

Neuromodulation therapy has been widely used in treating patients with DOC, divided into invasive and non-invasive neuromodulation according to the need for surgical assistance (Edlow et al., 2021; Shou et al., 2021; Wu et al., 2021). Non-invasive neuromodulation mainly includes repetitive transcranial magnetic stimulation (rTMS), transcranial direct current stimulation (tDCS), and transcutaneous auricular vagus nerve stimulation (taVNS).

Although taVNS is still in the early stages of scientific research and clinical application, thus far, it has been recognized as safe, convenient, inexpensive, and easy to use, allowing patients to use it from home and thus reducing their daily expenses. Therefore, it has been gaining increasing interest from clinicians and researchers.

To date, relevant studies have shown that taVNS is feasible and safe for patients with DOC. In 2017, our team reported a case of a 73-year-old female patient with the recovery of consciousness from VS to MCS after 4 weeks of treatment with taVNS and CRS-R score increased from 6 to 13. Functional MRI (fMRI) results showed that the patient had increased functional connectivity directly between the posterior cingulate/precuneus, hypothalamus, thalamus, ventral medial prefrontal cortex, and superior temporal gyrus, and decreased functional connectivity between the posterior cingulate and precuneus and cerebellum (Yu et al., 2017). In our opinion, taVNS enhances functional connectivity in the default mode network of the patient’s brain, which may be the main reason for the recovery of consciousness. Our previous study found that the retention of auditory stimuli was important for the response to taVNS in patients with DOC (Yu et al., 2021).

In their study, Hakon et al. (2020) treated 5 patients diagnosed with VS or MCS with taVNS for 8 weeks and found improvement in consciousness in 3 out of the 5 patients, with 2 patients progressing from VS and MCS to eMCS and 1 patient progressing from VS to MCS. In another study, 14 DOC patients (VS = 6, MCS = 8) received taVNS in the left ear for only 4 weeks. One of the MCS patients showed new signs of consciousness at the end of the 4-week stimulation, and the other four showed new signs of consciousness at the 4-week follow-up time point (Noé et al., 2020).

In the above study, only one patient experienced intermittent ear pruritus during stimulation; however, no significant relationship with taVNS was found, indicating that taVNS is a feasible and safe treatment for patients with DOC.

Although taVNS has shown positive results in studies of DOC, the overall effectiveness rate is low, and an understanding of the mechanisms of consciousness regulation is lacking. A growing body of evidence suggests that the optimal frequency of taVNS may vary across disorders. E.g., a clinical study of taVNS in therapeutic epilepsy showed that patients in the 25-Hz group had significantly lower seizure frequency compared to the 1-Hz group (Schnakers et al., 2008). In another clinical study of migraine patients, researchers found that although both 1 and 25 Hz taVNS improved the prognosis of patients with chronic migraine, a greater improvement was observed with 1 Hz taVNS (Schnakers et al., 2009). In their vagal cortical pathway model, Briand et al. (2020) suggested that brainstem activation may be critical for taVNS to lead to recovery of consciousness in patients with DOC. Several researchers evaluated brainstem fMRI responses to 2, 10, 25, and 100 Hz taVNS in healthy subjects and found that 100 Hz stimulation elicited the strongest brainstem response (Sclocco et al., 2020). Therefore, exploring the optimal stimulation frequency parameters for taVNS-treated should be addressed in order to improve the therapeutic efficacy of taVNS in patients with DOC.

We propose to preliminarily evaluate the optimal stimulation frequency parameters in patients with DOC treated with taVNS by changes in CRS-R and EEG before and after taVNS. The behavioral changes induced by taVNS can be measured by CRS-R, while changes in brain function brought about by taVNS can be measured by EEG. Based on previous studies, we believe that the increase in spectral power in the alpha and theta bands may represent an improvement in patient awareness (King et al., 2013; Chennu et al., 2014; Sitt et al., 2014). However, due to the small number of relevant studies, we mainly considered these two indicators but were not limited to them. Our previous study found that EEG changes were more pronounced in MCS patients than in VS patients before and after taVNS treatment (Yifei et al., 2022).

In this study, we will record the CRS-R scores and resting EEG of 15 MCS patients before and after five random taVNS at different frequencies (1, 10, 25, 50, and 100 Hz) and one sham stimulation. Changes in CRS-R scores will be used as the primary evaluation index, and the immediate effects of EEG will be used as a secondary index to assess the effects of different frequencies of taVNS on MCS patients. In this way, we will attempt to initially explore the optimal stimulation frequency parameters for taVNS treatment of DOC patients to optimize the neuromodulation paradigm and improve efficacy.

## Methods

### Patients

We plan to recruit 15 patients diagnosed with MCS at the Department of Neurosurgery, Beijing Tiantan Hospital, Capital Medical University. Patients will be assessed for inclusion criteria at admission by the principal investigator and screened according to exclusion criteria. Patients’ current state of consciousness will be determined using at least two CRS-R assessments. Inclusion and exclusion criteria are shown in Table 1.

**TABLE 1:** Study inclusion and exclusion criteria.

Inclusion criteria	Exclusion criteria
(1) Meeting the diagnostic criteria for MCS	(1) Open craniotomy
(2) Aged between 19 and 65 years old	(2) Brainstem injury
(3) Responding to auditory stimuli	(3) Pacemaker
(4) 3–12 months after injury	(4) Pregnant women
(5) Skull defect area less than one-third	(5) Metallic brain implants
(6) Family members agreed to sign an informed consent form	(6) Serious medical conditions that may affect clinical diagnosis or EEG activity

### Procedures

The pilot study will begin as early as day 30 after the patient’s injury and will be completed within 1 year of the injury. Written informed consent will be obtained from the patient’s legal representative following an open discussion of the study objectives, methods, and potential risks. The patient’s legal representative will be informed that the patient’s legal representative has the right to ask the patient to withdraw from the study at any time during the course of the study and that each party will retain a copy of the signed informed consent form.

**FIGURE 1:**
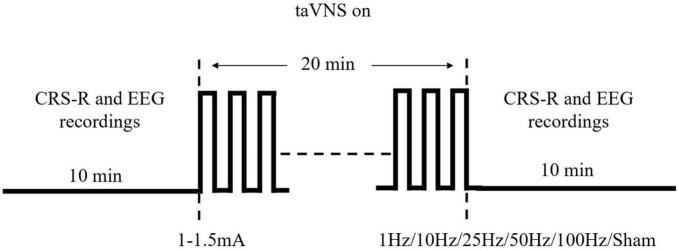
Single stimulation process and parameters used for stimulation.

This is a prospective exploratory trial including 15 MCS patients, each receiving a total of five different frequencies of taVNS (1, 10, 25, 50, and 100 Hz) and a sham stimulus as a blank control for 20 min, where the order of stimulation is completely randomized. We chose these five frequencies to span the range used in previous taVNS studies, including our own. Patient’s CRS-R scores will be recorded before and after each stimulation. Each CRS-R score will be performed by no fewer than two physicians with extensive clinical experience. Also, by using a bedside 32-channel EEG (Nicolet EEG V32, Natus Neurology, USA), a 10-min resting EEG will be recorded before and after each stimulation, respectively. The interval between stimulations will be at least 48 h. SDZ-IIB electronic stimulator (Suzhou Medical Supplies Factory) will be used to provide stimulation. The experimental flow is shown in Figure 1.

**FIGURE 2:**
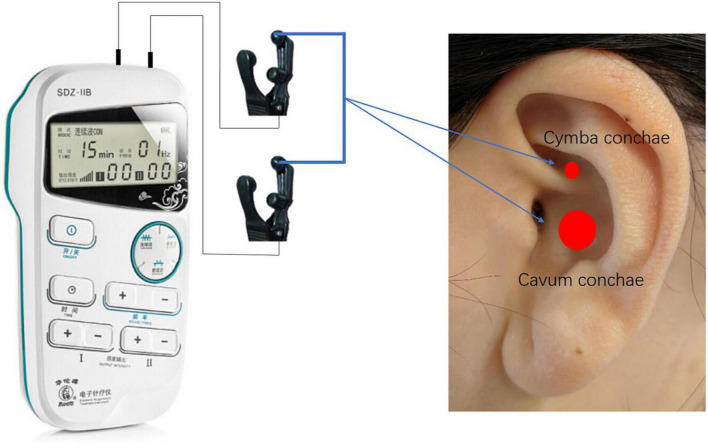
Stimulation sites of taVNS.

Consistent with our previous experiments, a pair of identical-looking clips will be placed on both ears. The clips are designed with three carbon-impregnated silicone tips, one of which serves as the common end for supporting the posterior surface of the auricle, and the other two tips are designed to stimulate two skin surface points, one in the outer ear and the other in the navicular bone, with the two silicone tips at the outer ear point placed in the Cymba conchae and the Cavum conchae, respectively, as these are the two areas where the vagus nerve is most densely distributed in the ear. As shown in Figure 2, the SDZ-IIB electronic stimulator provides electrical pulses and continuous waves with a current of 1–1.5 mA. Real stimuli with frequencies of 1, 10, 25, 50, and 100 Hz are used on five occasions throughout the experiment, while a sham stimulus with the turned-off stimulator will be used on one occasion.

### Assignment of interventions: Blinding

Throughout the trial, subjects and their legal representatives, caregivers on the medical team, and all researchers involved in patient assessment and statistical analysis will be blinded to the assignment of the intervention. During the trial, six envelopes of the same color, texture, and size will be placed in a box that will only be opened on one side. The investigator will randomly select an envelope and give it to a clinically experienced physician who will adjust the stimulation parameters for the patient according to the envelope instructions, and the extracted envelope will not be placed back in the box. This physician will not have any communication (including written, email, or verbal communication) with the investigator, the subject and his or her legal representative, the entire medical team, or the research staff involved in patient assessment and statistical analysis.

### EEG recording and processing

**FIGURE 3:**
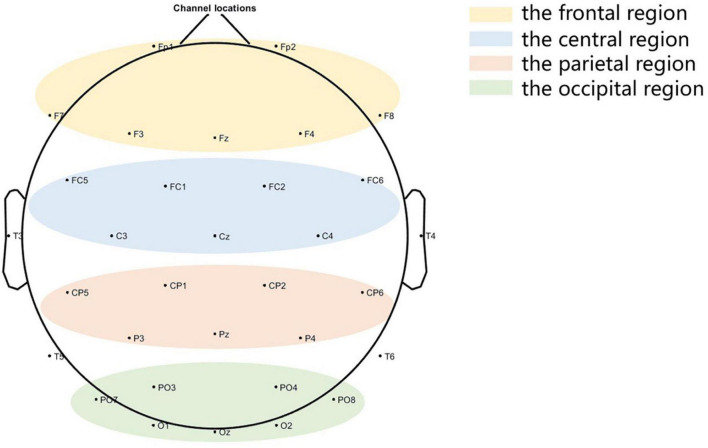
Brain local regions.

A total of 20 min of EEG signals will be recorded for each stimulation procedure, and 10 min will be acquired before and after taVNS. EEG will be acquired by 32 channels (Nicolet EEG V32, Natus Neurology, USA) at a sampling rate of 1000 Hz, and the device will have 32 Ag/AgCl electrodes based on the national standard 10–20 system setup. All electrodes will be used with FCz as the reference electrode and AFz as the ground electrode, and the skin/electrode impedance will be kept below 5 kΩ. Patients will be awake during the acquisition, and if they show signs of sleepiness, the CRS-R scale will be used to wake up the procedure, or the experiment will be paused. Thirty-two electrodes (Fp1, Fp2, F3, Fz, F4, F7, F8, FC1, FC2, FC5, FC6, C3, Cz, C4, CP1, CP2, CP5, CP6, Pz, P3, P4, PO3, PO4, PO7, PO8, O1, Oz, O2, T3, T4, T5, and T6) will be selected for offline EEG analysis, and the EEG display parameters will be set to trap 50 Hz and bandpass filtered to 1–40 Hz. The frontal, central, parietal, and occipital regions of the brain are shown in Figure 3.

### EEG analysis

The relative power of oscillations will be used to assess the effect of different frequencies of taVNS stimulation on the EEG of MCS patients (Bai et al., 2017). The relative power will be calculated as follows:


$$Relative\;Power\;(f_{1},f_{2})=Power\;(f_{1},f_{2})/Power\;(1,40)\;\times \;100\%,$$

where Power (*f*_1_,*f*_2_) indicates the absolute power between the low *f*_1_ and high *f*_2_ frequencies. Power (1,40) is the sum of power (1–40 Hz).

### taVNS and sham stimulations

The stimulation is provided by the SDZ-IIB electronic stimulator (Suzhou Medical Supplies Factory), which stably delivers continuous wave taVNS in the range of 1–100 Hz. In taVNS studies, the stimulation current intensity is commonly set according to the patient’s perception threshold, usually at 1–1.5 mA, depending on the patient’s tolerance (Badran et al., 2018). Based on previous experimental studies, we consider that the absence of a significant increase in heart rate, decrease in blood pressure, and painful expressions during stimulation mean that the patient tolerates the stimulation. Two silicone tips of the SDZ-IIB electronic stimulator are placed in the patient’s ear cavity and ear canal, and each patient receives five different frequencies of taVNS and a sham stimulus in a randomized order. The technical parameters of the sham stimulation are identical, but no current is passed through.

### Behavioral assessments

The CRS-R is the recommended method for classifying the level of consciousness (American Congress of Rehabilitation Medicine Brain Injury-Interdisciplinary Special Interest Group Disorders of Consciousness Task Force et al., 2010). The patient’s level of consciousness is assessed using the CRS-R. This scale is the most valid and sensitive method for identifying behavioral signs of consciousness, leading to a better diagnosis of UWS/VS and MCS. It consists of six following subscales: auditory, visual, motor, oral-motor and verbal functions, communication, and arousal level. There are 23 items ordered by complexity, ranging from reflexive to cognitively mediated behaviors (Giacino et al., 2004).

### Data collection and management

All information collected in this study will be kept confidential. All data will be stored using encryption measures, and paper copies will be stored electronically and encrypted. Subjects’ legal representatives will be informed of the nature of the collected data and their rights to that same data through specific information sheets.

### Statistical analysis

SAS 9.4 (SAS Institute, Cary, Cary, NC, USA) statistical software will be used, and data analysis will be completed by a third-party (Clinical Evaluation Center of the Chinese Academy of Traditional Chinese Medicine) statistician. Filling of missing values: multiple filling methods will be used. The regression filling method will be used for monotonic missing data of measures, and Monte Carlo filling method will be used for arbitrary missing data; the logistic regression filling method will be used for monotonic missing data of counts, and the FCS filling method will be used for arbitrary missing data; the regression analysis will be used for sensitivity analysis.

## Discussion

Although previous studies have reported a positive effect of taVNS in the treatment of DOC patients, the efficacy is not stable due to different neuromodulation paradigms. Given the urgent therapeutic needs of DOC patients and the promising therapeutic prospects of taVNS, the scientific questions of optimal frequency parameters of taVNS should be urgently addressed.

The vagus nerve is the strongest parasympathetic nerve in the autonomic nervous system, consisting of 80% afferent and 20% efferent fibers, and serves as a bridge between the brain and the body in both directions (Butt et al., 2020). Vagus nerve stimulation is performed by electrically stimulating the vagus nerve to regulate brain activity (Shi et al., 2013). The distribution of the vagus nerve in the ear branch is mainly concentrated in the auricular region, including the Cymba conchae and the Cavum conchae (He et al., 2012). Therefore, electrical stimulation of the ear branch can produce similar effects to classical vagus nerve stimulation (Fang et al., 2016) without inducing perioperative risks.

The results of several previous studies have shown that taVNS modulates or activates cortical and subcortical areas associated with conscious control, including the locus coeruleus, nucleus accumbens, hypothalamus, medial prefrontal cortex, dorsolateral prefrontal cortex, anterior cingulate cortex, and posterior cingulate cortex (Kraus et al., 2007; Ruffoli et al., 2011; Cao et al., 2017; Corazzol et al., 2017).

In our previous study, we reported the effect of taVNS on cerebral hemodynamics in patients with DOC (Yu et al., 2021). Among 10 DOC patients included in the study, 5 responded to auditory stimulation and 5 showed no response. After 4 weeks of taVNS treatment, patients who responded to auditory stimulation had increased cerebral blood flow in several brain regions, a significant increase in CRS-R scores, and GOS scores indicating a good prognosis. In contrast, in patients who did not respond to auditory stimulation, the increase in cerebral blood flow with taVNS treatment was relatively weak, with a significant increase only in the left cerebellum and no significant changes in CRS-R scores or GOS scores. Therefore, our team suggests that the preservation of auditory function may be a key factor in response to taVNS in patients with DOC.

Our recent study focused on the EEG changes in 12 DOC patients before and after 14 days of taVNS treatment (Yifei et al., 2022), revealing a decrease in δ-band energy and an increase in β-band energy in the EEG of MCS patients after treatment, while no significant changes were observed in VS. Therefore, in this experiment, we used the diagnosis of MCS and preservation of auditory function as important screening criteria in order to improve the success rate.

By reviewing six studies on tavNS for patients with DOC, Jang and Cho (2022) found that four studies reported positive outcomes in patients with DOC treated with taVNS, including two EEG studies and two fMRI studies. However, in terms of stimulation frequency, 20–25 Hz was chosen by most studies, probably because there were no studies on the optimal stimulation frequency of taVNS in patients with DOC. Accordingly, the FDA-approved frequency range of 20–30 Hz for vagus nerve stimulation ensued.

The range of stimulation frequencies along the vagus nerve is clearly lacking in consideration for taVNS. We found that different stimulation frequencies may be appropriate for taVNS in different diseases. Also, different frequencies for the same disease produce different therapeutic effects. E.g., Marsal et al. (2021) innovatively boosted the taVNS stimulator to 2 kHz for the treatment of rheumatoid arthritis disease, and after 12 weeks of treatment, the mean DAS28-CRP significantly decreased in 27 patients compared to baseline, demonstrating that a 100-fold higher treatment frequency was more effective in patients with rheumatoid arthritis than the conventional 20 Hz. In another study, 24 patients with migraine were randomized into two groups, one of which was treated with 1 Hz, and the control group was treated with 20 Hz, both for 4 weeks. The results showed that after 4 weeks of treatment, the efficacy of 1 Hz was significantly due to 20 Hz (Cao et al., 2021).

In contrast to the previous study, we will eliminate the sparse wave treatment and replace it with a continuous wave treatment. This is because the experiment needs to control for a single variable frequency. Based on previous reports in the literature, we will use 1 Hz, 10 Hz, 25 Hz, 100 Hz, and sham stimuli as controls and 50 Hz as a supplement considering the large gap between the 25 and 100 Hz ranges. According to the existing literature (Sun et al., 2021), the retention effect of taVNS on the EEG lasts for about 1 h. Therefore, we will set a 48-h interval, i.e., a 48-h interval before the two stimuli, to ensure that the next stimulus does not receive the effects of the previous one.

In this study, we propose investigating the changes in behavior and brain function induced by taVNS in DOC patients using CRS-R and EEG measurements. In addition, the relative power of the oscillations will be used to assess the effects of different frequencies of taVNS on the EEG of MCS patients. Based on this, we will attempt to initially explore the selection of the optimal stimulation frequency parameters of taVNS for DOC patients through this experiment and to lay the foundation for subsequent experimental studies.

## Data availability statement

The original contributions presented in this study are included in the article/supplementary material, further inquiries can be directed to the corresponding authors.

## Author contributions

WZ, HJ, JH, and PR contributed to the conception and design of the study. YZ, YY, JZ, and YFW reviewed the manuscript and suggested the changes. WZ wrote the first draft of the manuscript. YW, Y-NZ, and SZ embellished and revised the language of the manuscript. All authors participated in the revision of the manuscript, read, and approved the submitted version.
